# Effect of trial-to-trial variability on optimal event-related fMRI design: Implications for Beta-series correlation and multi-voxel pattern analysis

**DOI:** 10.1016/j.neuroimage.2015.11.009

**Published:** 2016-01-15

**Authors:** Hunar Abdulrahman, Richard N. Henson

**Affiliations:** a MRC Cognition & Brain Sciences Unit, Cambridge, England, United Kingdom; b University of Cambridge, Cambridge, United Kingdom

**Keywords:** fMRI design, General Linear Model, Bold variability, Least squares all, Least squares separate, MVPA, Trial based correlations

## Abstract

Functional magnetic resonance imaging (fMRI) studies typically employ rapid, event-related designs for behavioral reasons and for reasons associated with statistical efficiency. Efficiency is calculated from the precision of the parameters (Betas) estimated from a General Linear Model (GLM) in which trial onsets are convolved with a Hemodynamic Response Function (HRF). However, previous calculations of efficiency have ignored likely variability in the neural response from trial to trial, for example due to attentional fluctuations, or different stimuli across trials. Here we compare three GLMs in their efficiency for estimating average and individual Betas across trials as a function of trial variability, scan noise and Stimulus Onset Asynchrony (SOA): “Least Squares All” (LSA), “Least Squares Separate” (LSS) and “Least Squares Unitary” (LSU). Estimation of responses to individual trials in particular is important for both functional connectivity using “Beta-series correlation” and “multi-voxel pattern analysis” (MVPA). Our simulations show that the ratio of trial-to-trial variability to scan noise impacts both the optimal SOA and optimal GLM, especially for short SOAs < 5 s: LSA is better when this ratio is high, whereas LSS and LSU are better when the ratio is low. For MVPA, the consistency across voxels of trial variability and of scan noise is also critical. These findings not only have important implications for design of experiments using Beta-series regression and MVPA, but also statistical parametric mapping studies that seek only efficient estimation of the mean response across trials.

## Highlights

• Trial-wise connectivity and multi-voxel pattern analyses can use different GLMs.
• Trial-to-trial variability affects both the optimal GLM and optimal SOA.
• Optimal GLM and SOA depend on estimation of population or sample mean.
• Optimal GLM for single voxel analysis depends on ratio of trial- to scan-variability.
• Optimal GLM for pattern analysis depends on coherency of variability across voxels.

## Introduction

Many fMRI experiments use rapid presentation of trials of different types (conditions). Because the time between trial onsets (or Stimulus Onset Asynchrony, SOA) is typically less than the duration of the BOLD impulse response, the responses to successive trials overlap. The majority of fMRI analyses use linear convolution models like the General Linear Model (GLM) to extract estimates of responses to different trial-types (i.e., to deconvolve the fMRI response; Friston et al., 1998). The parameters of the GLM, reflecting the mean response to each trial-type, or even to each individual trial, are estimated by minimizing the squared error across scans (where scans are typically acquired with repetition time, or TR, of 1–2 s) between the timeseries recorded in each voxel and the timeseries that is predicted, based on i) the known trial onsets, ii) assumptions about the shape of the BOLD impulse response and iii) assumptions about noise in the fMRI data.

Many papers have considered how to optimize the design of fMRI experiments, in order to maximize statistical efficiency for a particular contrast of trial-types (e.g., Dale, 1999, Friston et al., 1999, Josephs and Henson, 1999). However, these papers have tended to consider only the choice of SOA, the probability of occurrence of trials of each type and the modeling of the BOLD response in terms of a Hemodynamic Response Function (HRF) (Henson, 2015, Liu et al., 2001). Few studies have considered the effects of variability in the amplitude of neural activity evoked from trial to trial (though see Josephs and Henson, 1999, Duann et al., 2002, Mumford et al., 2012). Such variability across trials might include systematic differences between the stimuli presented on each trial (Davis et al., 2014). This is the type of variability, when expressed differently across voxels, that is relevant to multi-voxel pattern analysis (MVPA), such as representational similarity analysis (RSA) (Mur et al., 2009). However, trial-to-trial variability is also likely to include other components such as random fluctuations in attention to stimuli, or variations in endogenous (e.g., pre-stimulus) brain activity that modulates stimulus-evoked responses (Becker et al., 2011, Birn, 2007, Fox et al., 2006); variability that can occur even for replications of exactly the same stimulus across trials. This is the type of variability utilized by trial-based measures of functional connectivity between voxels (so-called “Beta-series” regression, Rissman et al., 2004).

**Fig. 1:**
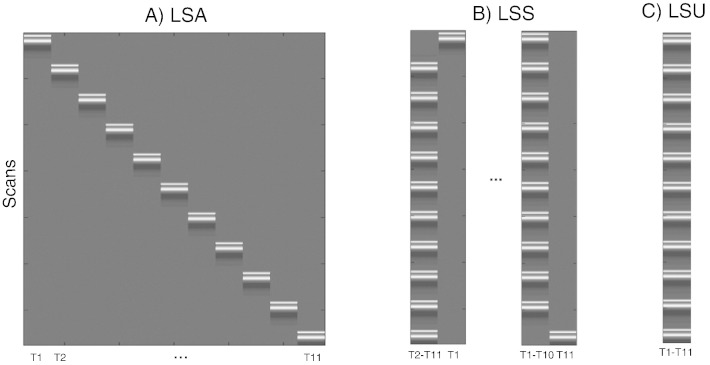
Design matrices for (A) LSA (Least Squares-All), (B) LSS (Least Squares-Separate) and (C) LSU (Least Squares-Unitary). T(number) = Trial number.

If one allows for variability in the response across trials of the same type, then one has several options for how to estimate those responses within the GLM. Provided one has more scans than trials (i.e. the SOA is longer than the TR), and provided the HRF is modeled with single (canonical) shape (i.e., with one degree of freedom), one could model each trial as a separate regressor in the GLM (Fig. 1A). Mumford et al. (2012) called this approach “Least-Squares All” (LSA), in terms of the GLM minimizing the squared error across all regressors. Turner (2010) introduced an alternative called “Least-Squares Separate” (LSS; Fig. 1B). This method actually estimates a separate GLM for each trial. Within each GLM, the trial of interest (target trial) is modeled as one regressor, and all the other (non-target) trials are collapsed into another regressor. This approach has been promoted for designs with short SOAs, when there is a high level of collinearity between BOLD responses to successive trials (Mumford et al., 2012). For completeness, we also consider the more typical GLM in which all trials of the same type are collapsed into the same regressor, and call this model “Least-Squares Unitary” (LSU). Though LSU models do not distinguish different trials of the same type (and so trial variability is relegated to the GLM error term), they are used to estimate the mean response for each trial-type, and we show below that the precision of this estimate is also affected by the ratio of trial variability to scan noise.

In the current study, we simulated the effects of different levels of trial-to-trial variability, as well as scan-to-scan noise (i.e., noise), on the ability to estimate responses to individual trials, across a range of SOAs (assuming that neural activity evoked by each trial was brief – i.e., less than 1 s – and locked to the trial onset, so that it can be effectively modeled as a delta function). More specifically, we compared the relative efficiency of the three types of GLM – LSU, LSA and LSS – for three distinct questions: 1) estimating the population or sample mean of responses across trials, as relevant, for example, to univariate analysis of a single voxel (e.g., statistical parametric mapping), 2) estimating the response to each individual trial, as relevant, for example, to trial-based measures of functional connectivity between voxels (Rissman et al., 2004), and 3) estimating the pattern of responses across voxels for each trial, as relevant to MVPA (e.g., Mumford et al., 2012). In short, we show that different GLMs are optimal for different questions, depending on the SOA and the ratio of trial variability to scan noise.

## Methods

We simulated fMRI timeseries for a fixed scanning duration of 45 min (typical of fMRI experiments), sampled every TR = 1 s. We modeled events by delta functions that were spaced with SOAs in steps of 1 s from 2 s to 24 s, and convolved with SPM's (www.fil.ion.ucl.ac.uk/spm) canonical HRF, scaled to have peak height of 1. The scaling of the delta-functions (true parameters) for the first trial-type (at a single voxel) was drawn from a Gaussian distribution with a population mean of 3 and standard deviation (SD) that was one of 0, 0.5, 0.8, 1.6, or 3. Independent zero-mean Gaussian noise was then added to each TR, with SD of 0.5, 0.8, 1.6 or 3,^1^ i.e., producing amplitude SNRs of 6, 3.8, 1.9 or 1 respectively. (Note that, as our simulations below show, the absolute values of these standard deviations matter little; what matters is the ratio of trial variability relative to scan noise.)

For the simulations with two trial-types, the second trial-type had a population mean of 5. The two trial-types were randomly intermixed. For the simulations of two trial-types across two voxels, either the same sample of parameter values was used for each voxel (coherent trial variability), or different samples were drawn independently for each voxel (incoherent trial variability). The GLM parameters (“Betas”, **β**) were estimated by least-squares fit of each of the GLMs in Fig. 1:

$${\hat{\beta }}_{OLS}={\left(X^{T}X\right)}^{-1}X^{T}y$$

where **X**^*T*^ is the transpose of the GLM design matrix and **y** is a vector of fMRI data for a single voxel. In extra simulations, we also examined a L2-regularized estimator for LSA models (equivalent to ridge regression; see also Mumford et al., 2012):

$${\hat{\beta }}_{RLS}={\left(X^{T}X+\lambda I\right)}^{-1}X^{T}y$$

where **I** is a scan-by-scan identity matrix and *λ* is the degree of regularization, as described in the Discussion section. A final constant term was added to remove the mean BOLD response (given that the absolute value of the BOLD signal is arbitrary). The precision of these parameter estimates was estimated by repeating the data generation and model fitting N = 10,000 times. This precision can be defined in several ways, depending on the question, as detailed in the Results section. Note that for regularized estimators, there is also a bias (whose trade-off with efficiency depends on the degree of regularization), tending to shrink the parameter estimates towards zero, but we do not consider this bias here.

Note that we are only considering the accuracy of the parameter estimates across multiple realizations (simulations, e.g., sessions, participants, or experiments), e.g., for a “random-effects” group analysis across participants. We do not consider the statistical significance (e.g., T-values) for a single realization, e.g., for a “fixed effects” within-participant analysis. The latter will also depend on the nature of the scan-to-scan noise (e.g., which is often autocorrelated and dominated by lower-frequencies) and on the degrees of freedom (dfs) used in the GLM (e.g., a LSA model is likely to be less sensitive than an LSU model for detecting the mean trial-response against noise, since it leaves fewer dfs to estimate that noise). Nonetheless, some analysis choices for a single realization – such as the use of a high-pass filter to remove low-frequency noise (which is also applied to the model) – will affect the parameter estimates, as we note in passing.

In some cases, transients at the start and end of the session were ignored by discarding the first and last 32 s of data (32 s was the length of the canonical HRF), and only modeling trials whose complete HRF could be estimated. A single covariate of no interest was also then added to each GLM that modeled the initial and final “partial” trials. When a highpass filter was applied, it was implemented by a set of additional regressions representing a Discrete Cosine Transform (DCT) set capturing frequencies up to 1/128 Hz (the default option in SPM12).

Finally, we also distinguished two types of LSS model: in LSS-1 (as shown in Fig. 1), the non-target trials were modeled as a single regressor, independent of their trial-type. In the LSS-2 model, on the other hand, non-target trials were modeled with a separate regressor for each of the two trial-types (more generally, the LSS-N model would have N trial-types; Turner et al., 2012). This distinction is relevant to classification. The LSS-N model will always estimate the target parameter as well as or better than the LSS-1 model; however, the LSS-N model requires knowledge of the trial-types (class labels). If one were to estimate classification using cross-validation in which the training and test sets contained trials from the same session, the use of labels for LSS-N models would bias classification performance. In practice, training and test sets are normally drawn from separate sessions (one other reason being that this avoids the estimates being biased by virtue of sharing the same error term; see Mumford et al., 2014). However, we thought the distinction between LSS-1 and LSS-N models would be worth exploring in principle, noting that if one had to train and test with trials from the same session (e.g., because one had only one session), then the LSS-1 model would be necessary.^2^

## Results

### Question 1. Optimal SOA and GLM for estimating the average trial response

For this question, one wants the most precise (least variable) estimate of the mean response across trials (and does not care about the responses to individual trials; cf. Questions 2 and 3 below). There are at least two ways of defining this precision.

### Precision of Population Mean (PPM)

If one regards each trial as measuring the same “thing”, except for random (zero-mean) noise, then the relevant measure is the precision of the population mean (PPM):

$$PPM=\frac{1}{std_{i=1..N}\left(\sum_{j=1}^{M}\frac{{\hat{\beta }}_{ij}}{M}-\beta \right)}=\frac{1}{std_{i=1..N}\left(\sum_{j=1}^{M}\frac{{\hat{\beta }}_{ij}}{M}\right)}$$

where *std*_*i* = 1.. *N*_ is the standard deviation across *N* simulations and ${\hat{\beta }}_{ij}$ is the parameter estimate for the *j*-th of M trials in the *i*-th simulation. *β* is the true population mean (3 in simulations here), though as a constant, is irrelevant to PPM (cf. PSM measure below). Note also that, because the least-square estimators are unbiased, the difference between the estimated and true population mean will tend to zero as the number of scans/trials tends to infinity.

The PPM measure is relevant when each trial includes, for example, random variations in attention, or when each trial represents a stimulus drawn randomly from a larger population of stimuli, and differences between stimuli are unknown or uninteresting.

**Fig. 2:**
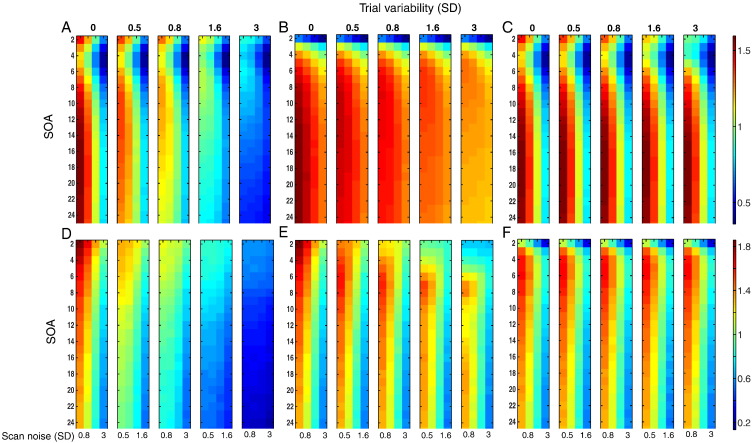
Efficiency for estimating mean of a single trial-type (top panels) or the mean difference between two trial-types (bottom panels) as a function of SOA and scan noise for each degree of trial variability. Panels A–C show results for a single-trial-type LSU model, using A) precision of population mean (PPM), B) PPM without transients, and C) precision of sample mean (PSM). Panels D–E show results for difference between two randomly intermixed trial-types, using D) PPM and E) PSM. Panel F shows corresponding PSM results but using LSA model (LSS gives similar results to LSU). The top number on each subplot represents the level of trial-to-trial variability, y-axes are the SOA ranges and x-axes are scan noise levels. The color map is scaled to base-10 logarithm, with more efficient estimates in hotter colors, and is the same for panels A–C (shown right top) and D–F (shown right bottom).

PPM is plotted against SOA and scan noise for estimating the mean of a single trial-type using the LSU model in Fig. 2A, where each sub-plot reflects a different degree of trial variability. Efficiency decreases as both types of variability increase, as expected since the LSU model does not distinguish these two types of variability. When there is no trial variability (leftmost sub-plot), the optimal SOAs are 17 s and 2 s. Optimal SOAs of approximately 17 s are consistent with standard results for estimating the mean response versus baseline using fixed-SOA designs (and correspond to the dominant bandpass frequency of the canonical HRF, Josephs and Henson, 1999). The second peak in efficiency for the minimal SOA simulated (2 s) is actually due to transients at the start and end of each session, and disappears when these transients are removed (Fig. 2B). The reason for this is given in Supplementary Fig. 3. The high efficiency at short-SOAs is also removed if the data and model are high-pass filtered (results very similar to Fig. 2B), as is common in fMRI studies to remove low-frequency noise. Nonetheless, some studies do not employ high-pass filtering because they only care about the parameter estimates (and not their associated error, as estimated from the scan noise; see the Methods section), in which case the peak at 2 s could be a reason to consider using short SOAs.

Another feature of Fig. 2B is that, as the trial variability increases across left-to-right sub-plots, the optimal SOA tends to decrease, for example from 17 s when there is no trial variability down to 6 s when the SD of trial variability is 3. The advantage of a shorter SOA is that more trials can be fit into the finite session, making it more likely that the sample mean of the parameters will be close to the population mean. Provided the trial variability is as large as, or greater than, scan noise, this greater number of trials improves the PPM. This effect of trial variability on optimal SOA has not, to our knowledge, been considered previously.

### Precision of Sample Mean (PSM)

**Supplementary Fig. 1:**
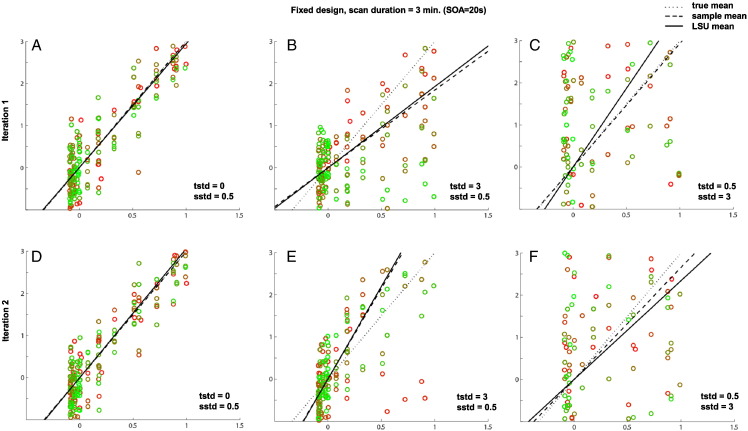
Effects of trial variability and scan noise on efficiency of estimating the mean of single trial-type using LSU when SOA = 20 s. tstd = trial beta standard deviation, sstd = scan noise standard deviation. The scatter plots show simulated BOLD activity (y-axis) against LSU's regressor (x-axis) for a 3 min session sampled at TR = 1 s, after removing the transients in the first and last 32 s. The black solid line is the linear least-squares fit, whose slope corresponds to the LSU parameter estimate; the slope of the dashed line corresponds to the sample mean; the slope of the dotted line corresponds to the true (population) mean (here equal to 3). The more closely the dots fall to the best-fit line, the more accurate the estimation of the slope of that line. The closer the solid line to the dotted line, the more precise the estimate of the population mean (i.e., PPM in main paper). Note that there are the same number of dots (scans) in each panel, which are bunched into vertical columns, where the number of columns corresponds to the SOA and the number of dots per column corresponds to the number of trials. The dot colors change smoothly from green to red in proportion to the proximity to the beginning and the end of the session (i.e., dots with similar hue are close together in time). In comparison to the first column (with only a small amount of scan noise), it can be seen that the LSU estimate (solid line) deviates more from the true mean (dotted line) as the trial-variability (middle column) or scan noise (bottom column) increases, as in Fig. 2B of the paper. The top and bottom panels indicate two simulations with different samples of trial variability and scan noise. The difference between PPM and PSM can be seen by comparing top and bottom panels. In panels B and E, for example, though the LSU estimate (solid line) differs from the true mean (dotted line), it closely tracks the sample mean (dashed line). This is why the PSM does not decease with increasing trial variability in Fig. 2C of the paper.

If one only cares about the particular stimuli presented in a given session (i.e., assumes that they fully represent the stimulus class), and assumes that each trial is noise-free realization of a stimulus, then a more appropriate measure of efficiency is the Precision of Sample Mean (PSM):

$$PSM=\frac{1}{std_{i=1..N}\left(\sum_{j=1}^{M}\frac{{\hat{\beta }}_{ij}}{M}-\sum_{j=1}^{M}\frac{\beta_{ij}}{M}\right)}=\frac{1}{std_{i=1..N}\left(\sum_{j=1}^{M}\frac{{\hat{\beta }}_{ij}-\beta_{ij}}{M}\right)}$$

where *β*_*ij*_ is the true parameter for the *j*-th of M trials in the *i*-th simulation. Fig. 2C shows the corresponding values of PSM for a single trial-type under the LSU model. The most striking difference from Fig. 2A and B is that precision does not decrease as trial variability increases, because the sample mean is independent of the sample variance (see Supplementary Fig. 1). The other noticeable difference is the fact that the optimal SOA no longer decreases as the trial variability increases (it remains around 17 s for all levels of scan- and trial variability), because there is no longer any gain from having more trials with which to estimate the (sample) mean.

### Estimating difference between two trial-types

Whereas Fig. 2A–C present efficiency for estimating the mean response to a single trial-type versus baseline, Fig. 2D–F present efficiency for estimating the difference in mean response between two, randomly intermixed trial-types (see the Methods section). Note also that the results for intermixed trials are little affected by removing transients or high-pass filtering (see Supplementary Fig. 3).

The most noticeable difference in the PPM shown Fig. 2D, compared to Fig. 2A, is that shorter SOAs are always optimal, consistent with standard efficiency theory (Friston et al., 1999, Dale, 1999; see Supplementary Fig. 3). Fig. 2E shows results for PSM. As for a single trial-type in Fig. 2C, PSM no longer decreases with increasing trial variability as rapidly as does PPM, since trial variability is no longer a source of noise. Interestingly though, the optimal SOA also increases from 2 s with no trial variability (as for PPM) to 8 s with a trial SD of 3. This is because it becomes more difficult to distinguish trial variability from scan noise at low SOAs, such that scan noise can become misattributed to trial variability. Longer SOAs help distinguish these two types of variability, but very long SOAs (e.g., the second peak in Fig. 2E around 17 s) become less efficient for randomized designs (compared to fixed SOA designs, as in Fig. 2C) because the signal (now the differential response between trial-types) moves into lower frequencies and further from the optimal bandpass frequency of the HRF (Josephs and Henson, 1999). For further explanation, see the Supplementary material. However, when using the LSA model rather than the LSU model (Fig. 2F), trial variability can be better distinguished from scan noise, the optimal SOA is stable at around 6 s, and most importantly, PSM is better overall for high trial variability relative to LSU in Fig. 2E. We return to this point in the next section (see also Supplementary Fig. 4).

Note also that results in Fig. 2 for PPM and PSM using LSS-1/LSS-2 are virtually identical to those using LSU, since the ability to estimate the mean response over trials does not depend on how target and non-target trials are modeled (cf. Questions 2 and 3).

### Comparison of models

**Fig. 3:**
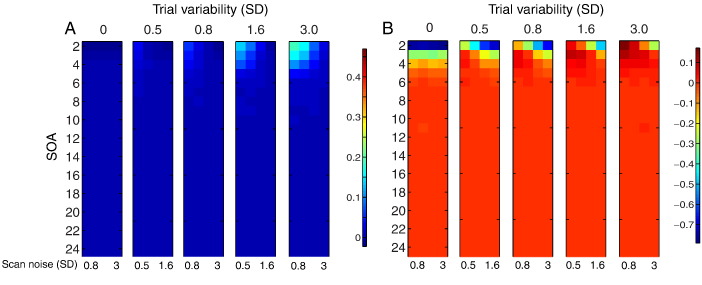
Log of ratio of PPM for LSA relative to LSU models for (A) estimating mean of a single trial-type, or (B) the mean difference between two randomly intermixed trial-types, as a function of SOA and scan noise for each degree of trial variability. The color maps are scaled to base-10 logarithm. See Fig. 2 legend for more details.

Fig. 3 shows the ratio of PPMs for LSA relative to LSU (the results for the ratio of PSMs are quantitatively more pronounced but qualitatively similar). For a single trial-type (Fig. 3A), LSA is more efficient than LSU when trial variability is high and scan noise is low. For the contrast of two randomly intermixed trial-types (Fig. 3B), LSA is again more efficient when trial variability is high and scan noise is low, though is now much less efficient when trial variability is low and scan noise is high. These results are important because they show that, even if one only cares about the mean response across trials (as typical for univariate analyses), it can be better to model each trial individually (i.e., using LSA), compared to using the standard LSU model, in situations where the trial variability is likely to be higher than the scan noise, and the SOA is short.

### Question 2. Optimal SOA and GLM for estimating individual trial responses in a single voxel

For this question, one wants the most precise estimate of the response to each individual trial, as necessary for example for trial-based connectivity estimation (Rissman et al., 2004).

### Precision of Sample Correlation (PSC)

In this case, a simple metric is the Precision of Sample Correlation (PSC), defined as:

$$PSC=\sum_{i=1}^{N}\frac{cor_{j}\left({\hat{\beta }}_{ij},\beta_{ij}\right)}{N}$$

where *cor*(*x*, *y*) is the sample (Pearson) correlation between *x* and *y*. Note that the LSU model cannot be used for this purpose, and PSC is not defined when the trial variability is zero (because *β*_*ij*_ is constant). Note also that there is no difference between a single trial-type and multiple trial-types in this situation (since each trial needs to be estimated separately).

**Fig. 4:**
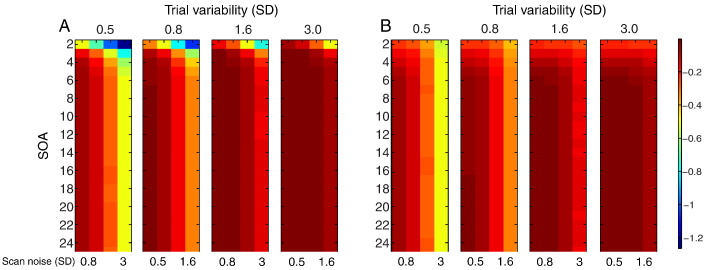
Log of precision of Sample Correlation (PSC) for two randomly intermixed trial-types for LSA (A) and LSS-1 (B). See Fig. 2 legend for more details.

PSC is plotted for LSA and LSS-1 in Fig. 4. For LSA, SOA had little effect as long as it was greater than 5 s when scan noise was high. For LSS-1, the optimal SOA was comparable, though shorter SOAs were less harmful for low trial variability. These models are compared directly in the next section.

### Comparison of models

**Fig. 5:**
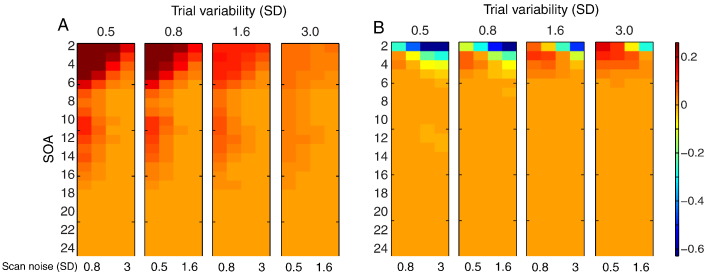
Log of ratio of PSC in Fig. 4 for (A) LSS-2 relative to LSS-1 and (B) LSA relative to LSS-2. See Fig. 2 legend for more details.

The ratio of PSC for LSS-2 to LSS-1 models is shown in Fig. 5A. As expected, distinguishing non-target trials by condition (LSS-2) is always better, particularly for short SOAs and low ratios of trial variability to scan noise. Fig. 5B shows the more interesting ratio of PSC for LSA relative to LSS-2. In this case, for short SOAs, LSA is better when the ratio of trial variability to scan noise is high, but LSS is better when the ratio of trial variability to scan noise is low. It is worth considering the reason for this in a more detail.

**Fig. 6:**
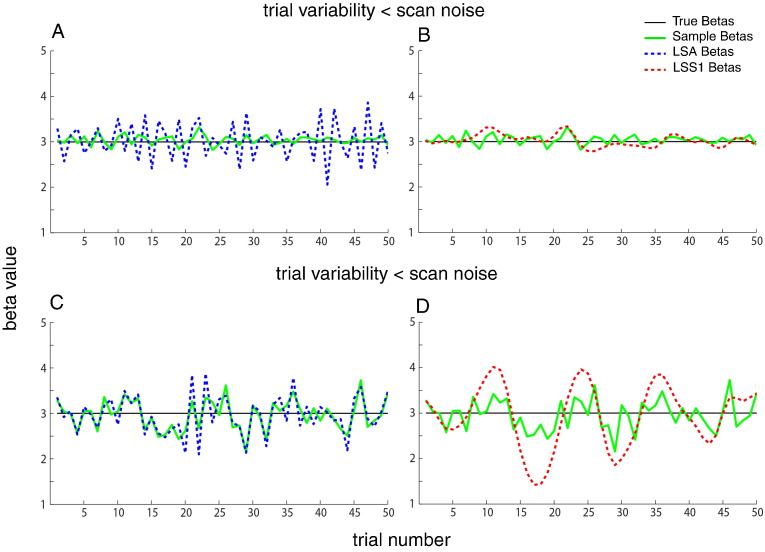
Example of sequence of parameter estimates (${\hat{\beta }}_{j}$) for 50 trials of one stimulus class with SOA of 2 s (true population mean B = 3) when trial variability (SD = 0.3) is greater than scan noise (SD = 0.1; top row) or trial variability (SD = 0.1) is less than scan noise (SD = 0.3; bottom row), from LSA (left panels, in blue) and LSS (right panels, in red). Individual trial responses *β*_*j*_ are shown in green (identical in the left and right plots).

The reason is exemplified in Fig. 6, which shows examples of true and estimated parameters for LSA and LSS for a single trial-type when the SOA is 2 s. The LSA estimates (in blue) fluctuate more rapidly across trials than do the LSS estimates (in red) — i.e., LSS forces temporal smoothness across estimates. When scan noise is greater than trial variability (top row), LSA “overfits” the scan noise (i.e., attributes some of the scan noise to trial variability, as mentioned earlier). In this case, the “regularized” LSS estimates are superior. However, when trial variability is greater than scan noise (bottom row), LSS is less able to track rapid changes in the trial responses, and LSA becomes a better model.

### Question 3. Optimal SOA and GLM for estimating pattern of individual trial responses over voxels

For this question, one wants the most precise estimate of the relative pattern across voxels of the responses to each individual trial, as relevant to MVPA (Davis et al. 2014).

### Classification performance (CP)

For this question, our measure of efficiency was classification performance (CP) of a support-vector machine (SVM), which was fed the pattern for each trial across two voxels. Classification was based on two-fold cross-validation, after dividing the scans into separate training and testing sessions. Different types of classifiers may produce different overall CP levels, but we expect the qualitative effects of SOA, trial variability and scan noise to be the same.

In the case of multiple voxels, there may be spatial correlation in the trial variability and/or scan noise, particularly if the voxels are contiguous. We therefore compared variability that was either fully coherent or incoherent across voxels, factorially for trial variability and scan noise. In the case of coherent trial variability, for example, the response for a given trial was identical across voxels, whereas for incoherent trial variability, responses for each voxel were drawn independently from the same Gaussian distribution. Coherent trial variability may be more likely (e.g., if levels of attention affect responses across all voxels in a brain region), though incoherent trial variability might apply if voxels respond to completely independent features of the same stimulus. In practice there may be a non-perfect degree of spatial correlation across voxels in both trial variability and scan noise, but by considering the two extremes we can interpolate to intermediate cases.

**Fig. 7:**
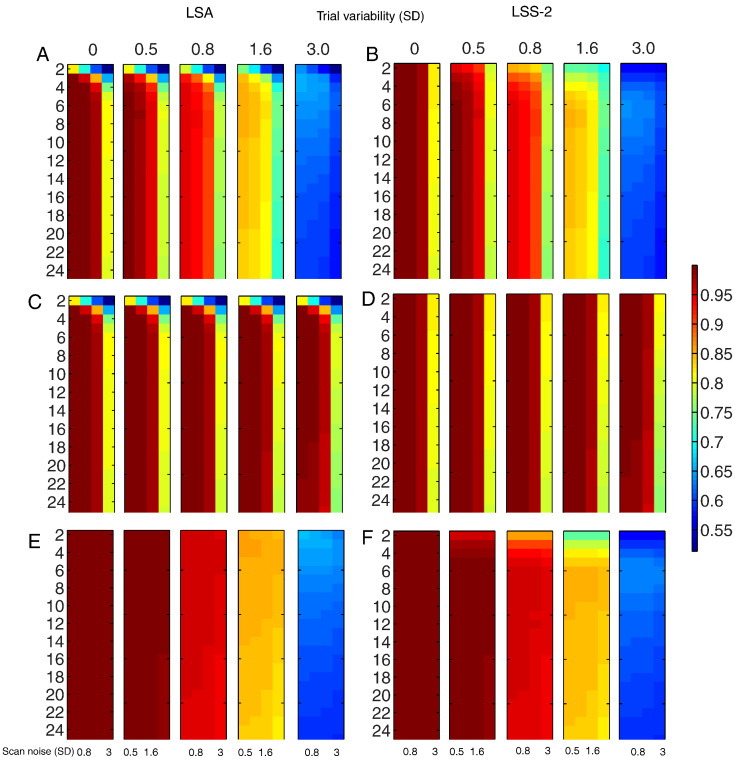
SVM classification performance for LSA (panels A + C + E) and LSS-2 (panels B + D + F) for (A) incoherent trial variability and incoherent scan noise (panels A + B), coherent trial variability and incoherent scan noise (panels C + D), and incoherent trial variability and coherent scan noise (panels E + F). Note color bar is not log-transformed (raw accuracy, where 0.5 is chance and 1.0 is perfect). Note that coherent and incoherent cases are equivalent when trial variability is zero (but LSA and LSS are not equivalent even when trial variability is zero). See Fig. 2 legend for more details.

Fig. 7 shows CP for incoherent trial variability and incoherent scan noise (top row), coherent trial variability and incoherent scan noise (middle row) and incoherent trial variability and coherent scan noise (bottom row), for LSA (left) and LSS-2 (right).^3^ When scan noise is incoherent (i.e., comparing top and middle rows), the most noticeable effect of coherent relative to incoherent trial variability was to maintain CP as trial variability increased, while the most noticeable effect of LSS-2 relative to LSA was to maintain CP as SOA decreased. The most noticeable effect of coherent relative to incoherent scan noise (when trial variability was incoherent, i.e., comparing top and bottom rows) was that CP decreased as trial variability increased, with little effect of scan noise levels, while the most noticeable effect of LSS-2 relative to LSA was to actually reduce CP as SOA decreased. In short, making trial variability or scan noise coherent across voxels minimizes the effects of the size of that type of variability on CP, because CP only cares about relative patterns across voxels.

When trial variability and scan noise are both incoherent (top row), the SOA has little effect for LSA and LSS-2 when trial variability is low (as long as SOA is more than approximately 5 s in the case of LSA), but becomes optimal around 3–8 s as trial variability increases. With coherent trial variability and incoherent scan noise (middle row), SOA has little effect for low scan noise (again as long as SOA is not too short for LSA), but becomes optimal around 6–8 s for LSA, or 2 s for LSS-2, when scan noise is high. With incoherent trial variability and coherent scan noise (bottom row), the effect of SOA for LSA was minimal, but for LSS-2, the optimal SOA approached 6–7 s with increasing trial variability.^4^ The reason for these different sensitivities of LSA and LSS to coherent versus incoherent trial variability is explored in the next section.

### Comparison of models

**Fig. 8:**
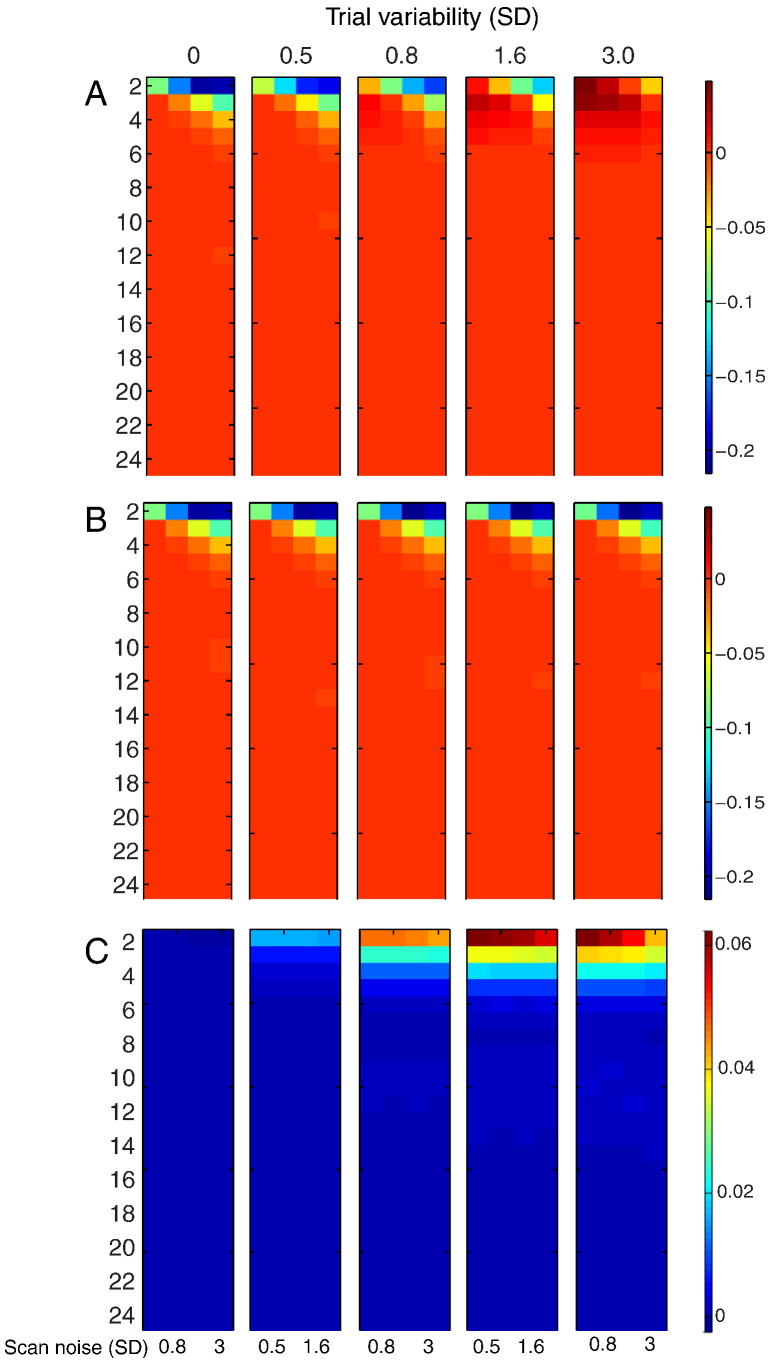
Log of ratio of LSA relative to LSS-2 SVM classification performance in Fig. 7 for (A) incoherent trial variability and incoherent scan noise, (B) coherent trial variability and incoherent scan noise and (C) incoherent trial variability and coherent scan noise. Note that coherent and incoherent cases are equivalent when trial variability is zero. See Fig. 2 legend for more details.

Fig. 8 shows the (log) ratio of CP for LSA relative to LSS-2 for the three rows in Fig. 7. Differences only emerge at short SOAs. For incoherent trial variability and incoherent scan noise (Fig. 8A), LSS-2 is superior when the ratio of trial variability to scan noise is low, whereas LSA is superior when the ratio of trial variability to scan noise is high, much like in Fig. 5B. For coherent trial variability and incoherent scan noise (Fig. 8B), on the other hand, LSS-2 is as good as, or superior to LSA (for short SOAs), when coherent trial variability dominates across the voxels (i.e., the LSA:LSS-2 ratio never exceeds 1, i.e. the log ratio never exceeds 0). For incoherent trial variability and coherent scan noise (Fig. 8C), LSA is as good as, or superior to LSS-2 (for short SOAs), particularly when trial variability is high and scan noise low.

**Fig. 9:**
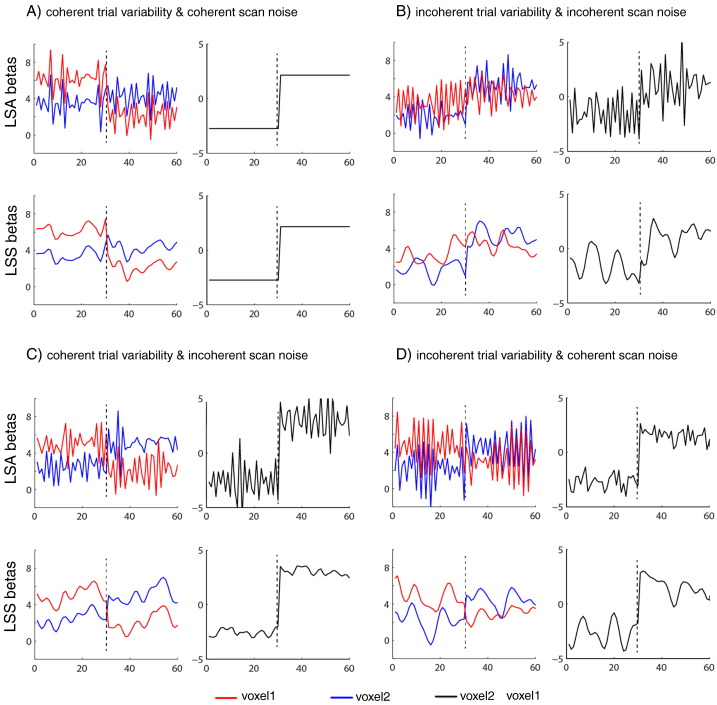
Panels A, B, C and D show different variations of coherency of trial-to-trial variability and scan noise across two voxels (SOA = 2 s and both trial SD and scan SD = 0.5). In each Panel, plots on left show parameters/estimates for 30 trials of each of two trial-types: true parameters (*β*_*j*_) for trials 1–30 are 5 and 3 for voxel 1 and voxel 2 respectively, while true parameters (*β*_*j*_) for trials 31–60 are 3 and 5 for voxel 1 and voxel 2 respectively. Plots on right show difference between voxels for each trial (which determines CP). Upper plots in each panel show corresponding parameter estimates (${\hat{\beta }}_{j}$) from LSA model; lower plots show estimates from LSS-2 model.

The reason for the interaction between LSA/LSS model and coherent/incoherent trial variability and scan noise (at short SOA) is illustrated in Fig. 9. The top plots in Panels A–D show LSA estimates, whereas the bottom plots show LSS estimates. The left plots show individual trial estimates, while the right plots show the difference between voxels for each trial, which determines the relative pattern across voxels and hence CP. For the special case where both scan noise and -trial variability are coherent across the voxels, as shown in Fig. 9A, the effects of both scan noise and trial variability are identical across voxels, so the difference between voxel 1 and voxel 2 allows perfect classification (CP = 100%). Panel B shows the opposite case where both scan noise and trial variability are incoherent (independent across the voxels), so neither type of variability cancels out across the voxels. This means that the relative performance of LSA to LSS performance depends on the ratio of scan noise to trial variability, similar to our findings for single voxel efficiency in Fig. 5B. Panel C shows the more interesting case of coherent trial variability across the voxels, which cancel out when we take the difference between voxel 1 and voxel 2, leaving only the scan noise, and hence LSS is always a better model regardless of the ratio of trial variability to scan noise. Panel D shows the complementary case where coherent scan noise cancels when taking the difference across the voxels, leaving only the trial variability, and hence LSA is always a better model.

## Discussion

Previous studies of efficient fMRI design have given little consideration to the effect of trial-to-trial variability in the amplitude of the evoked response. This variability might be random noise, such as uncontrollable fluctuations in a participant's attention, or systematic differences between the stimuli presented each trial. Through simulations, we calculated the optimal SOA and type of GLM (LSU vs LSA vs LSS) for three different types of research question. We summarize the main take-home messages, before considering other details of the simulations.

### General advice

There are three main messages for the fMRI experimenter:

1. If you only care about the mean response across trials of each type (condition), and wish to make inferences across a number of such means (e.g., one mean per participant), then while you might normally only consider the LSU model, there are situations where the LSA model is superior (and superior to LSS). These situations are when the SOA is short and the trial variability is higher than the scan noise (Fig. 3). Note however that when scan noise is less than trial variability, the LSA model will be inferior.
2. If you care about the responses to individual trials, for example for functional connectivity using Beta-series regression (Rissman et al., 2004), and your SOA is short, then whether you should use the typical LSA model, or the LSS model, depends on the ratio of trial variability to scan noise: in particular, when scan noise is higher than trial variability, the LSS model will do better (Fig. 5B).
3. If you care about the pattern of responses to individual trials across voxels, for MVPA, then whether LSA or LSS is better depends on whether the trial variability and/or scan noise is coherent across voxels. If trial variability is more coherent than scan noise, then LSS is better; whereas if scan noise is more coherent then trial variability, then LSA is better (Fig. 8).

As well as these main messages, our simulations can also be used to choose the optimal SOA for a particular question and contrast of trial-types, as a function of estimated trial variability and scan noise (using Fig. 2, Fig. 4, Fig. 7).

### Unmodeled trial variability

Even if trial-to-trial variability is not of interest, the failure to model it can have implications for other analyses, since this source of variance will end up in the GLM residuals. For example, analyses that attempt to estimate functional connectivity independent of trial-evoked responses (e.g., Fair et al., 2007) may end up with connectivity estimates that include unmodeled variations in trial-evoked responses, rather than the desired background/resting-state connectivity. Similarly, models that distinguish between item-effects and state-effects (e.g., Chawla et al., 1999) may end up incorrectly attributing to state differences what are actually unmodeled variations in item effects across trials. Failure to allow for trial variability could also affect comparisons across groups, e.g., given evidence to suggest that trial-to-trial variability is higher in older adults (assuming little difference in scan noise, Baum and Beauchamp, 2014).

Strictly speaking, unmodeled trial variability invalidates LSU for statistical inference within-participant (across-scans). LSA models overcome this problem, but at the cost of using more degrees of freedom in the model, hence reducing the statistical power for within-participant inference. In practice however, assuming trial variability is random over time, the only adverse consequence of unmodeled variance will be to increase temporal autocorrelation in the error term (within the duration of the HRF), which can be captured by a sufficient order of auto-regressive noise models (Friston et al., 2002). Moreover, this unmodeled variance does not matter if one only cares about inference at the level of parameters (with LSA) or level of participants (eg with LSU).

### Estimating the population vs sample mean

Our simulations illustrate the important difference between the ability to estimate the population mean across trials versus the sample mean. As can be seen in Fig. 2 (and Supplementary figures), the optimal SOA for our PPM and PSM metrics changes dramatically as a function of trial variability. The basic reason is that increasing the total number of trials, by virtue of decreasing the SOA, improves the estimate of the population mean (PPM), but is irrelevant to the sample mean (PSM). As noted in the Introduction, the question of whether one cares about PPM or PSM depends on whether the trials (e.g., stimuli) are a subset drawn randomly from a larger population (i.e., trial amplitude is a random effect), or whether the experimental trials fully represent the set of possible trials (i.e., trial amplitude is a fixed effect).

This sampling issue applies not only to the difference between PPM and PSM for estimating the mean across trials; it is also relevant to MVPA performance. If one estimates classification accuracy over all trials within a session, then all that matters is the precision of estimating the sample mean for that session, whereas if one estimates classification accuracy using cross-validation (i.e., training on trials in one session but testing on trials in a different session), then what matters is the precision of estimating the population mean. Moreover, if one is estimating responses to two or more trial-types within a session, then using separate regressors for the non-target trials of each condition (i.e., what we called the LSS-N model) is effectively using knowledge of the class labels, and so would bias classification performance. More generally however, it is advisable to perform cross-validation across sessions to ensure that training and test data are independent (Mumford et al., 2014), as we did here, in which case LSS-N is an appropriate (unbiased) model.

### Estimating individual trials: LSS vs LSA

Since the introduction of LSS by Turner (2010) and Mumford et al. (2012), it is becoming adopted in many MVPA studies. LSS effectively imposes a form of regularization of parameter estimates over time, resulting in smoother Beta series. This makes the estimates less prone to scan noise, which can help trial-based functional connectivity analyses too. However, as shown in Fig. 6, this temporal regularization also potentially obscures differences between nearby trials when the SOA is short (at which point LSA can become a better model). Thus for short SOA, the real value of LSS for functional connectivity analysis will depend on the ratio of trial variability to scan noise. This temporal regularization does not matter so much for MVPA analyses however, if the trial variability is coherent across voxels, because the resulting patterns across voxels become even more robust to (independent) scan noise across voxels, as shown in Fig. 9C.

We also considered a regularized least-square estimation of the parameters for LSA models (“L2-norm”; see the Methods section). The resulting estimates are not shown here because they were very similar to those from an LSS-1 model, i.e., showed temporal smoothing over time as in Fig. 6. This is important because, assuming the degree of regularization (*λ* for ${\hat{\beta }}_{RLS}$ equation in the Methods section) is known, L2-regularization is computationally much simpler than the iterative fitting required for LSS. Moreover, the degree of regularization is a free (hyper)parameter that could also be tuned by the user, for example as a function of the scan noise and its degree of spatial coherency. Thus in future we expect regularized versions of LSA will be preferred over LSS, at least for a single trial-type models (LSS may still offer more flexibility when more than one trial-type is distinguished, such as the LSS-2 models considered here). Other types of LSA regularization (e.g., using L1 rather than L2 norms, or even an explicit temporal smoothness constraint) may also be worth exploring in future, potentially increasing efficiency at the expense of bias (Mumford et al., 2012).

However, when scan noise is more coherent across voxels than is trial variability, LSA is better than LSS, even when the ratio of scan noise to trial variability is high. This is because the coherent fluctuations of scan noise cancel each other across the voxels, leaving only trial variability, which can be modeled better by LSA than LSS, as shown in Fig. 9D. It is difficult to predict which type of variability will be more coherent across voxels in real fMRI data. One might expect trial variability to be more coherent across voxels within an ROI, if, for example, it reflects global changes in attention (and the fMRI point-spread function / intrinsic smoothness is smaller than the ROI). This may explain why Mumford et al. (2012) showed an advantage of the LSS model in their data.

### Caveats

The main question for the experimenter is how to know the relative size of trial variability and scan noise in advance (and their degree of coherency across voxels, if one is interested in MVPA). If one only cared about which GLM is best, one could collect some pilot data, fit both LSA and LSS models, and compare the ratio of standard deviations of Betas across trials from these two models. This will give an indication of the ratio of trial variability to scan noise, and hence which model is likely to be best for future data (assuming this ratio does not change across session, participant, etc.). If one also wanted to know the optimal SOA, once could collect pilot data with a long SOA, estimate individual trials with LSA, and then compare the standard deviation of Betas across trials with the standard deviation of the scan error estimated from the residuals (assuming that the HRF model is sufficient). The Betas will themselves include a component of estimation error coming from scan noise, but this would at least place an upper bound on trial variability, from which one could estimate the optimal SOA for the main experiment. A better approach would be to fit a single, hierarchical linear (mixed effects) model that includes parametrization of both trial variability and scan noise (estimated simultaneously using maximum likelihood schemes, e.g., Friston et al., 2002), and use these estimates to inform optimal design for subsequent experiments. Note however that, if some of the trial variability comes from variations in attention, then the conclusions may not generalize to designs that differ in SOA (i.e., trial variability may actually change with SOA).

In the present simulations, we have assumed temporally uncorrelated scan noise. In reality, scan noise is temporally auto-correlated, and the GLM is often generalized with an auto-regressive (AR) noise model (in conjunction with high-pass filter) to accommodate this (e.g., Friston et al., 2002). Moreover, trial-to-trial variability seems likely to be temporally auto-correlated (e.g., owing to waxing and waning of sustained attention), which may improve the efficiency of LSS (given its temporal smoothing in Fig. 6). Regarding the spatial correlation in scan noise across voxels (for MVPA), this is usually dominated by haemodynamic factors like draining vessels and cardiac and respiratory signals, which can be estimated comparing residuals across voxels, or using external measurements. Future work could explore the impact on efficiency of such colored noise sources (indeed, temporal and spatial covariance constraints could also be applied to the modeling of trial variability in hierarchical models, Friston et al., 2002). Future studies could also explore the efficiency of non-random designs, such as blocking trial-types in order to benefit estimators like LSS.

Finally, there are more sophisticated modeling approaches than the common GLM, some of which have explicitly incorporated trial variability, using maximum likelihood estimation of hierarchical models mentioned above (e.g., Brignell et al., 2015), or nonlinear optimization of model parameters (e.g. Lu et al., 2005). Nonetheless, the general principles of efficiency, i.e., how best to estimate trial-level parameters, should be the same as outlined here.

The following are the supplementary data related to this article.

**Supplementary Fig. 2:**
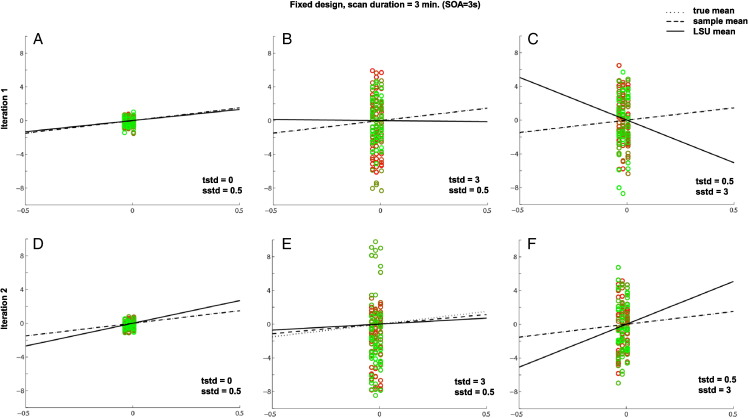
Effects of trial variability and scan noise on efficiency of estimating the mean of single trial-type using LSU when SOA = 3 s. See legend of Supplementary Fig. 1 for more details. Effects of trial variability and scan noise on efficiency of estimating the mean of single trial-type using LSU when SOA = 3 s. See legend of Supplementary Fig. 1 for more details. When SOA is short, the spread of points along the x-axis reduces compared to Supplementary Fig. 1 (where SOA = 20 s), and this reduced range prevents precise estimate of the slope of the best-fitting line, which is why PPM generally decreases as SOA decreases in Fig. 2B of main paper (without the influence of transients; cf Supplementary Fig. 3). When SOA is short, the spread of points along the x-axis reduces compared to Supplementary Fig. 1 (where SOA = 20 s), and this reduced range prevents precise estimate of the slope of the best-fitting line, which is why PPM generally decreases as SOA decreases in Fig. 2B of main paper (without the influence of transients; cf Supplementary Fig. 3). A more subtle point is that, when trial-variability is high and scan noise low (Panels B and E), the LSU estimate (solid line) can be closer to the true mean (dotted line), on average across simulations, than when the SOA is longer (cf. Panels B and E of Supplementary Fig. 1). This is because a shorter SOA entails more trials in total, i.e., a greater number of columns, and better “anchoring” the best-fitting line. This explains why the optimal SOA for PPM decreases as trial-variability increases in Fig. 2B of main paper. Note that the sample mean is independent of the number of trials, so the optimal SOA does not change this way for PSM in Fig. 2C of main paper. A more subtle point is that, when trial-variability is high and scan noise low (Panels B and E), the LSU estimate (solid line) can be closer to the true mean (dotted line), on average across simulations, than when the SOA is longer (cf. Panels B and E of Supplementary Fig. 1). This is because a shorter SOA entails more trials in total, i.e., a greater number of columns, and better “anchoring” the best-fitting line. This explains why the optimal SOA for PPM decreases as trial-variability increases in Fig. 2B of main paper. Note that the sample mean is independent of the number of trials, so the optimal SOA does not change this way for PSM in Fig. 2C of main paper.

**Supplementary Fig. 3:**
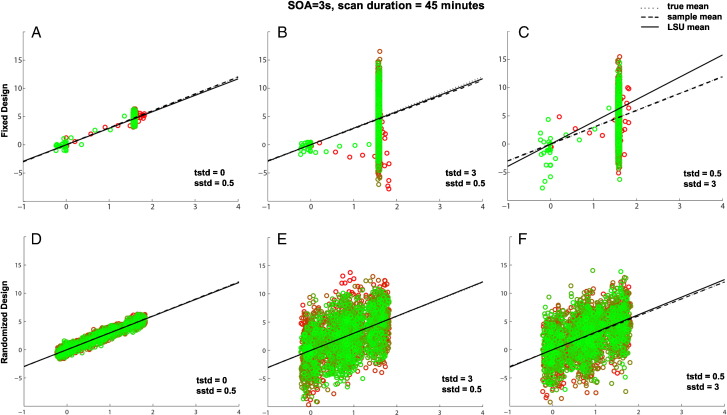
Effects of transients on efficiency of estimating a single trial-type (top row) and efficiency for two randomly-intermixed trial-types (bottom row) when SOA = 3 s. See legend of Supplementary Fig. 1 for more details. Effects of transients on efficiency of estimating a single trial-type (top row) and efficiency for two randomly-intermixed trial-types (bottom row) when SOA = 3 s. See legend of Supplementary Fig. 1 for more details. Transients at the start and end of the session, which correspond to the second, smaller cluster of points near x = 0 in the top row, help to stabilize the slope of the regression line, relative to Supplementary Fig. 2, even though only representing a small fraction of the total number of scans (the session is now 45 min long). This explains the second peak at short SOA in Fig. 2A (but not Fig. 2B) of main paper. Transients at the start and end of the session, which correspond to the second, smaller cluster of points near x = 0 in the top row, help to stabilize the slope of the regression line, relative to Supplementary Fig. 2, even though only representing a small fraction of the total number of scans (the session is now 45 min long). This explains the second peak at short SOA in Fig. 2A (but not Fig. 2B) of main paper. The SOA in the lower panels is a randomized intermixed design of two trial-types, but one of which has a mean and standard deviation of 0 (equivalent to a “null event” in the terminology of Josephs and Henson, 1999). This jittering dramatically increases efficiency (cf. Panel D with Supplementary Figs. 1A and 2A), consistent with standard efficiency theory, which explains the optimally short SOA in Fig. 2D of the main paper. Note also that transients also have little effect in such designs. The SOA in the lower panels is a randomized intermixed design of two trial-types, but one of which has a mean and standard deviation of 0 (equivalent to a “null event” in the terminology of Josephs and Henson, 1999). This jittering dramatically increases efficiency (cf. Panel D with Supplementary Figs. 1A and 2A), consistent with standard efficiency theory, which explains the optimally short SOA in Fig. 2D of the main paper. Note also that transients also have little effect in such designs.

**Supplementary Fig. 4:**
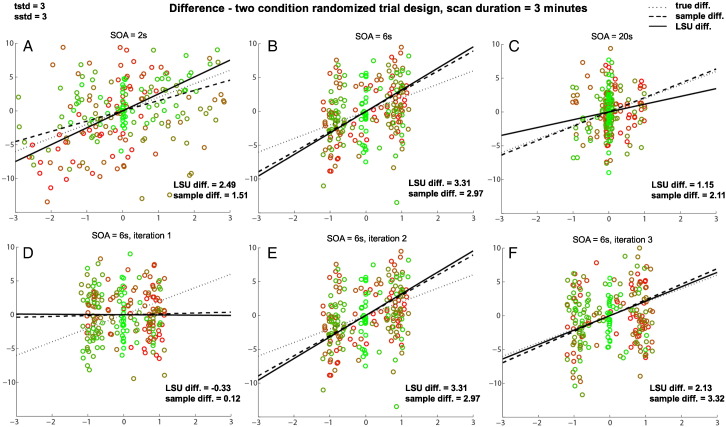
Effects of SOA on PSM for difference between two randomly-intermixed trial-types for three different SOAs (top row) and three different iterations when SOA = 6 s (bottom row) when trial variability plus scan noise are high. See legend of Supplementary Fig. 1 for more details. Effects of SOA on PSM for difference between two randomly-intermixed trial-types for three different SOAs (top row) and three different iterations when SOA = 6 s (bottom row) when trial variability plus scan noise are high. See legend of Supplementary Fig. 1 for more details. In this case, one is interested in the best estimate of the difference in slopes for the two trial-types, regardless of whether the slope of each trial-type is itself estimated efficiently. If the scan noise is low (not shown), the difference between trial-types can be estimated best for shortest SOAs (like in Supplementary Fig. 3), even though the slopes of individual trial-types are estimated better when SOA is longer (explaining the difference in optimal SOA for Fig. 2B versus 2E in main paper, and consistent with different optimal SOAs for [1 − 1] and [1 1] contrasts in Josephs and Henson, 1999). In this case, one is interested in the best estimate of the difference in slopes for the two trial-types, regardless of whether the slope of each trial-type is itself estimated efficiently. If the scan noise is low (not shown), the difference between trial-types can be estimated best for shortest SOAs (like in Supplementary Fig. 3), even though the slopes of individual trial-types are estimated better when SOA is longer (explaining the difference in optimal SOA for Fig. 2B versus 2E in main paper, and consistent with different optimal SOAs for [1 − 1] and [1 1] contrasts in Josephs and Henson, 1999). However, unlike the jittered SOA shown in bottom row of Supplementary Fig. 3, both trial-types now have non-zero trial variabilities. This means that when the scan noise is high (as shown here), it becomes difficult to distinguish differences between trial-types from random variations in scan noise. This makes estimation of the true difference in slopes worse, as shown in Fig. 2B in main paper. Estimation of the difference in sample means, however, is optimal for intermediate SOAs (like SOA = 6 s here). The reason for this is that at these SOAs, the scans (points) fall into two clumps near the ends of the best-fit line, which serve to better “anchor” that line. This is shown in the bottom row, with three different random samples of data at SOA = 6 s, where the LSU estimate tracks the sample difference, even though both vary considerably from the population difference (owing to the high trial variability). This explains the peak around SOA = 6 s with high trial variability for PSM in Fig. 2E of the main paper, but not for PPM in Fig. 2D. However, unlike the jittered SOA shown in bottom row of Supplementary Fig. 3, both trial-types now have non-zero trial variabilities. This means that when the scan noise is high (as shown here), it becomes difficult to distinguish differences between trial-types from random variations in scan noise. This makes estimation of the true difference in slopes worse, as shown in Fig. 2B in main paper. Estimation of the difference in sample means, however, is optimal for intermediate SOAs (like SOA = 6 s here). The reason for this is that at these SOAs, the scans (points) fall into two clumps near the ends of the best-fit line, which serve to better “anchor” that line. This is shown in the bottom row, with three different random samples of data at SOA = 6 s, where the LSU estimate tracks the sample difference, even though both vary considerably from the population difference (owing to the high trial variability). This explains the peak around SOA = 6 s with high trial variability for PSM in Fig. 2E of the main paper, but not for PPM in Fig. 2D.

**Supplementary Fig. 5:**
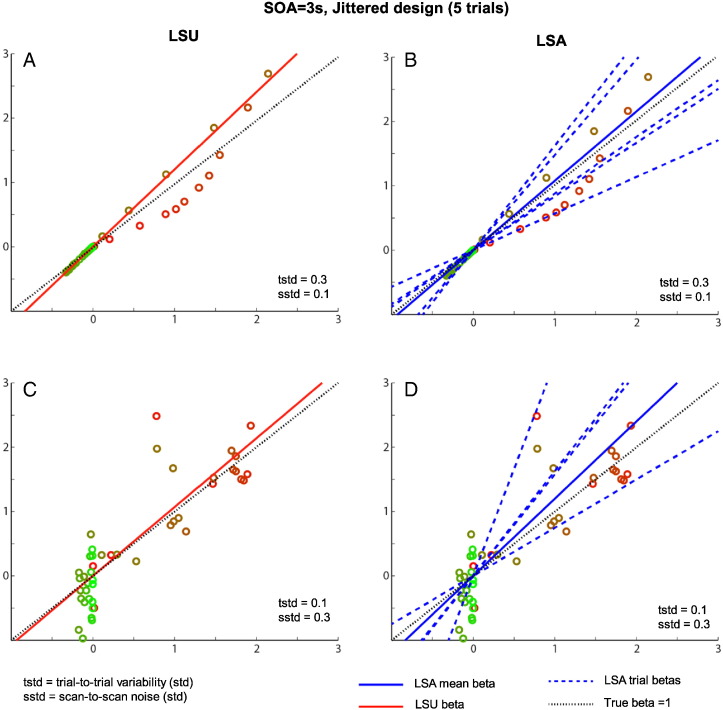
Comparison between LSU (left panels) and LSA (right panels) for a short SOA (3 s) jittered design of only 5 trials, with either low (top row) or higher (bottom row) scan noise. See legend of Supplementary Fig. 1 for more details, though note that true (population) beta is now 1, for illustration purposes. Comparison between LSU (left panels) and LSA (right panels) for a short SOA (3 s) jittered design of only 5 trials, with either low (top row) or higher (bottom row) scan noise. See legend of Supplementary Fig. 1 for more details, though note that true (population) beta is now 1, for illustration purposes. When trial-to-trial variability is higher than the scan noise, as in the upper row, the LSU model in Panel A cannot account for all the variability from trial-to-trial, and some trials (brown colored points) have a greater effect on the best-fitting slope than other trials (red points). The LSA model, on the other hand, can effectively fit a different line to each trial (dashed blue lines), as shown in Panel B, producing a better overall fit (and less affected by more extreme points). When averaging the slopes (Betas) from each trial, one can obtain a more precise estimate of the population mean, as in Fig. 2F of the main paper. However, when scan noise is higher than the trial-to-trial variability, as in the bottom row, LSA's extra flexibility (higher degrees of freedom) in Panel D means that some of its individual trial estimates fit the scan noise instead. This results in LSA over-fitting the data, and hence its estimate of the population mean (averaging across trials) now becomes less precise than for the more constrained LSU fit in Panel A. Together, this explains why the relative advantage of LSU vs LSA (at short SOA) depends on the ratio of trial variability to scan noise, as shown in Fig. 3 of the main paper.

## Footnotes

1 Note that in the special case of zero trial variability and zero scan noise, all parameters would be estimated perfectly, and so all GLMs are equivalent.

2 An alternative would be to block trials of each type within the session, rather than randomly intermix them as assumed here, and ensure that blocks are separated by at least the duration of the HRF, such that estimates of each trial were effectively independent (ignoring any autocorrelations in the scan noise). However in this case, the distinction between LSS-1 and LSS-N also becomes irrelevant.

3 The case of coherent trial variability and coherent scan noise is not shown, because CP is then perfect (and LSS and LSA are identical), for reasons shown in Fig. 9.

4 Note that we have assumed that trial-types were intermixed randomly within the same session. One could of course have multiple sessions, with a different trial-type in each session, and perform classification (e.g., cross-validation) on estimates across sessions. In this case, the relevant efficiency results would resemble those for a single trial-type shown in Fig. 2A.

## References

[bb0010] Baum S.H., Beauchamp M.S. (2014). Greater BOLD variability in older compared with younger adults during audiovisual speech perception. PLoS ONE.

[bb0015] Becker R., Reinacher M., Freyer F., Villringer A., Ritter P. (2011). How ongoing neuronal oscillations account for evoked fMRI variability. J. Neurosci..

[bb0020] Birn R.M. (2007). The behavioral significance of spontaneous fluctuations in brain activity. Neuron.

[bb0025] Brignell C.J., Browne W.J., Dryden I.L., Francis S.T. (2015). Mixed Effect Modelling of Single Trial Variability in Ultra-high Field fMRI. http://ArXiv%20preprint,%20ArXiv:%201501.05763.

[bb0030] Chawla D., Rees G., Friston K.J. (1999). The physiological basis of attentional modulation in extrastriate visual areas. Nat. Neurosci..

[bb0035] Dale A.M. (1999). Optimal experimental design for event-related fMRI. Hum. Brain Mapp..

[bb0040] Davis T., LaRocque K.F., Mumford J.A., Norman K.A., Wagner A.D., Poldrack R.A. (2014). What do differences between multi-voxel and univariate analysis mean? How subject-, voxel-, and trial-level variance impact fMRI analysis. NeuroImage.

[bb9100] Duann J.R., Jung T.P., Kuo W.J., Yeh T.C., Makeig S., Hsieh J.C., Sejnowski T.J. (2002). Single-trial variability in event-related BOLD signals. Neuroimage.

[bb0045] Fair D.A., Schlaggar B.L., Cohen A.L., Miezin F.M., Dosenbach N.U.F., Wenger K.K., Fox M.D., Snyder A.Z., Raichle M.E., Petersen S.E. (2007). A method for using blocked and event-related fMRI data to study “resting state” functional connectivity. NeuroImage.

[bb0050] Fox M.D., Snyder A.Z., Zacks J.M., Raichle M.E. (2006). Coherent spontaneous activity accounts for trial-to-trial variability in human evoked brain responses. Nat. Neurosci..

[bb0055] Friston K.J., Fletcher P., Josephs O., Holmes A., Rugg M.D., Turner R. (1998). Event-related fMRI: characterizing differential responses. NeuroImage.

[bb0060] Friston K.J., Zarahn E., Josephs O., Henson R.N., Dale A. (1999). Stochastic designs in event-related fMRI. Neuroimage.

[bb0065] Friston K.J., Glaser D.E., Henson R.N.A., Kiebel S., Phillips C., Ashburner J. (2002). Classical and Bayesian inference in neuroimaging: applications. NeuroImage.

[bb0075] Henson R.N., Toga Arthur W. (2015). Design efficiency. Brain Mapping: An Encyclopedic Reference.

[bb0080] Josephs O., Henson R.N.A. (1999). Event-related functional magnetic resonance imaging: modelling, inference and optimization. Philos. Trans. R. Soc. Lond. B Biol. Sci..

[bb0085] Liu T.T., Frank L.R., Wong E.C., Buxton R.B. (2001). Detection power, estimation efficiency, and predictability in event-related fMRI. NeuroImage.

[bb0090] Lu Y., Jiang T., Zang Y. (2005). Single-trial variable model for event-related fMRI data analysis. IEEE Trans. Med. Imaging.

[bb0095] Mumford J.A., Davis T., Poldrack R.A. (2014). The impact of study design on pattern estimation for single-trial multivariate pattern analysis. NeuroImage.

[bb0100] Mumford J.A., Turner B.O., Ashby F.G., Poldrack R.A. (2012). Deconvolving BOLD activation in event-related designs for multivoxel pattern classification analyses. NeuroImage.

[bb0105] Mur M., Bandettini P.A., Kriegeskorte N. (2009). Revealing representational content with pattern-information fMRI—an introductory guide. Soc. Cogn. Affect. Neurosci..

[bb0115] Rissman J., Gazzaley A., D'Esposito M. (2004). Measuring functional connectivity during distinct stages of a cognitive task. NeuroImage.

[bb0120] Turner B.O. (2010). Comparison of methods for the use of pattern classification on rapid event-related fMRI data. Poster Session Presented at the Annual Meeting of the Society for Neuroscience; San Diego, CA.

[bb0125] Turner B.O., Mumford J.A., Poldrack R.A., Ashby F.G. (2012). Spatiotemporal activity estimation for multivoxel pattern analysis with rapid event-related designs. NeuroImage.

